# Effects of the Co-Occurrence of Diabetes and Tooth Loss on Cognitive Function

**DOI:** 10.1093/geroni/igab046.793

**Published:** 2021-12-17

**Authors:** Huabin Luo, Chenxin Tan, Brenda Plassman, Frank Sloan, Mark Schwartz, Samrachana Adhikari, Xiang Qi, Bei Wu

**Affiliations:** 1 East Carolina University, Greenville, North Carolina, United States; 2 New York University, New York, New York, United States; 3 Duke University, Durham, North Carolina, United States; 4 New York University, NYC, New York, United States; 5 Rory Meyers College of nursing, New York, New York, United States

## Abstract

Using data from the 2006, 2012, and 2018 waves of the Health and Retirement Study, we estimated effects of co-occurrence of diabetes mellitus (DM) and complete tooth loss (CTL), both self-reported, on cognitive function among 10,816 adults age 50+. Cognitive function was measured using a shortened version of the Telephone Interview for Cognitive Status. Results from the fixed effects linear regression model show that in comparison to those with neither condition, adults having both DM and CTL had the worst cognitive function (b = 1.49, p < 0.001), followed by having CTL alone (b = 0.78, p < 0.001), and having DM alone (b = 0.42, p < 0.001). Our study suggests that CTL is a stronger risk factor for lower cognitive function than DM, and the co-occurrence of DM and CTL poses additive risk. Further research is needed to investigate the pathway from DM and CTL to poor cognition.

